# Preoperative prediction of lymphovascular invasion of colorectal cancer by radiomics based on 18F-FDG PET-CT and clinical factors

**DOI:** 10.3389/fradi.2023.1212382

**Published:** 2023-08-08

**Authors:** Yan Yang, Huanhuan Wei, Fangfang Fu, Wei Wei, Yaping Wu, Yan Bai, Qing Li, Meiyun Wang

**Affiliations:** ^1^ Department of Medical Imaging, People’s Hospital of Zhengzhou University, Henan Provincial People’s Hospital, Zhengzhou, China; ^2^ Henan Key Laboratory of Neurological Imaging, Henan Provincial People’s Hospital, Zhengzhou, China

**Keywords:** colorectal cancer, lymphovascular invasion, PET-CT, radiomics, preoperative prediction

## Abstract

**Purpose:**

The purpose of this study was to investigate the value of a clinical radiomics model based on Positron emission tomography-computed tomography (PET-CT) radiomics features combined with clinical predictors of Lymphovascular invasion (LVI) in predicting preoperative LVI in patients with colorectal cancer (CRC).

**Methods:**

A total of 95 CRC patients who underwent preoperative ^18^F-fluorodeoxyglucose (FDG) PET-CT examination were retrospectively enrolled. Univariate and multivariate logistic regression analyses were used to analyse clinical factors and PET metabolic data in the LVI-positive and LVI-negative groups to identify independent predictors of LVI. We constructed four prediction models based on radiomics features and clinical data to predict LVI status. The predictive efficacy of different models was evaluated according to the receiver operating characteristic curve. Then, the nomogram of the best model was constructed, and its performance was evaluated using calibration and clinical decision curves.

**Results:**

Mean standardized uptake value (SUVmean), maximum tumour diameter and lymph node metastasis were independent predictors of LVI in CRC patients (*P* < 0.05). The clinical radiomics model obtained the best prediction performance, with an Area Under Curve (AUC) of 0.922 (95%CI 0.820–0.977) and 0.918 (95%CI 0.782–0.982) in the training and validation cohorts, respectively. A nomogram based on the clinical radiomics model was constructed, and the calibration curve fitted well (*P* > 0.05).

**Conclusion:**

The clinical radiomics prediction model constructed in this study has high value in the preoperative individualized prediction of LVI in CRC patients.

## 1. Introduction

At present, the annual new cases of colorectal cancer (CRC) rank the third among malignant tumors, and the death cases rank the second among cancer deaths (1, 2). At present, surgery remains the main treatment for early-stage CRC, but studies have shown that 20%–30% of patients suffer from recurrence or metastasis after surgery (3). Because the prognosis of recurrent CRC is generally poor, identifying reliable prognostic factors is crucial. Lymphovascular invasion (LVI) reflects the distant metastasis of cancer cells, in which cancer cells first enter the circulatory system and spread to the entire body through lymph or blood vessels. Many studies have shown that LVI is an independent risk factor or subclinical stage of lymph node metastasis (4, 5), and once lymph node metastasis occurs, it is classified as stage III rectal cancer (6), and the treatment plan is also changed. Therefore, the prediction and identification of LVI before stage III is of great clinical significance for the establishment of individualized treatment plans.

Compared with CT images, high-resolution magnetic resonance imaging (MRI) can accurately evaluate extramural vascular invasion with a large vessel diameter (diameter ≥3 mm), but it has low sensitivity for the diagnosis of some early intramural invasion and extramural small vessel invasion (7, 8). The application of radiomics quantitative analysis can mine more high-dimensional quantitative features than CT and MRI images and make accurate predictions by constructing models (9, 10). Previous studies on lymph vessels of CRC mostly used CT images or MRI sequences, while the application of PET radiomics features in CRC mainly focused on the prediction of tumour gene mutations, presence or absence of distant metastasis, prognosis prediction and efficacy evaluation (11–13). This study aims to evaluate the value of a clinical radiomics model based on PET-CT imaging features combined with clinical independent predictors of LVI in the preoperative prediction of LVI in rectal cancer patients. It can be used as an important reference for clinical treatment decision-making.

## 2. Materials and methods

### 2.1. Patient selection

Ethical approval for this retrospective study was obtained by the Medical Ethics Committee of Henan Provincial People's Hospital [approval number: (2019) No. 68], and the need for written informed consent was waived.

This was a retrospective single-centre study. A total of 239 patients with CRC confirmed by postoperative pathology in Henan Provincial People's Hospital from January 2016 to May 2022 were retrospectively collected. Inclusion criteria were: (1) CRC was confirmed by postoperative pathology, (2) PET/CT was performed within 1 month before surgery, (3) complete preoperative PET-CT images were available. Exclusion criteria were: (1) preoperative neoadjuvant therapy (radiotherapy, chemotherapy or targeted therapy), (2) vascular invasion was not clear in pathology, and (3) combined with other pelvic malignant tumours or poor image quality could not be evaluated.

Clinical information of patients was obtained by searching medical records, including age, sex, serum levels of tumour markers: carcinoembryonic antigen (CEA ≤ 5 ng/ml), carbohydrate antigen 199 (CA199 ≤ 35U/ml), carbohydrate antigen125 (CA125 ≤ 35U/ml), and tumor differentiation degree. Maximum standardized uptake value (SUVmax), mean standardized uptake value (SUVmean), peak standardized uptake value (SUVpeak), metabolic tumor volume (MTV), total lesion glycolysis (TLG), maximum tumour diameter and lymph node metastasis were obtained by PET-CT information.

### 2.2. Histopathology

All patients underwent surgical resection after ^18^F-FDG PET-CT examination. All surgical specimens were reviewed by pathologists with more than 6 years of experience in abdominopelvic pathology, specifically to assess for the presence of LVI. LVI was diagnosed when tumour cells or tumour cell thrombi were observed within an endothelium-lined space or if tumour cells had destroyed a lymphovascular wall, as described in a previous study (14).

### 2.3. PET-CT image acquisition

The imaging equipment used was a Discovery™ VCT PET-CT instrument from GE Company in the United States. ^18^F-FDG was synthesized by a GE Minitrace medical cyclotron and FDG automatic synthesis device (Beijing Patte Biotechnology Co., LTD.). The radiochemical purity of ^18^F-FDG was >95%. High-performance liquid chromatography was used for quality control. Fingertip blood glucose was collected before the examination, and the blood glucose level was controlled below 11.1 mmol/l. The patients fasted for 6–8 h before the examination, and the dose of ^18^F-FDG was 5.55 MBa/kg. After the injection, the patients rested for 40–50 min in a quiet and suitable temperature environment before imaging. The bladder was emptied, and the stomach was filled with 500 ml of water. PET and CT scanning were performed from the head to the feet. CT images were acquired with the following scanning parameters: tube voltage, 120 kV; tube current, 170 mA; field of view, 40 mm; slice thickness, 3.75 mm; tube speed, 0.6 ms/r; pitch, 0.984 mm; scanning time, 13.8 s; and matrix, 256 × 256. Three-dimensional PET scans were performed with 4–6 window levels according to height, and each window level took 3 min to scan. After CT attenuation correction and iterative reconstruction, the fused images of the coronal, sagittal and transverse planes were obtained by Integrated Registration software in the GE 4.6ADW postprocessing station.

### 2.4. PET-CT image analysis

**Figure 1:**
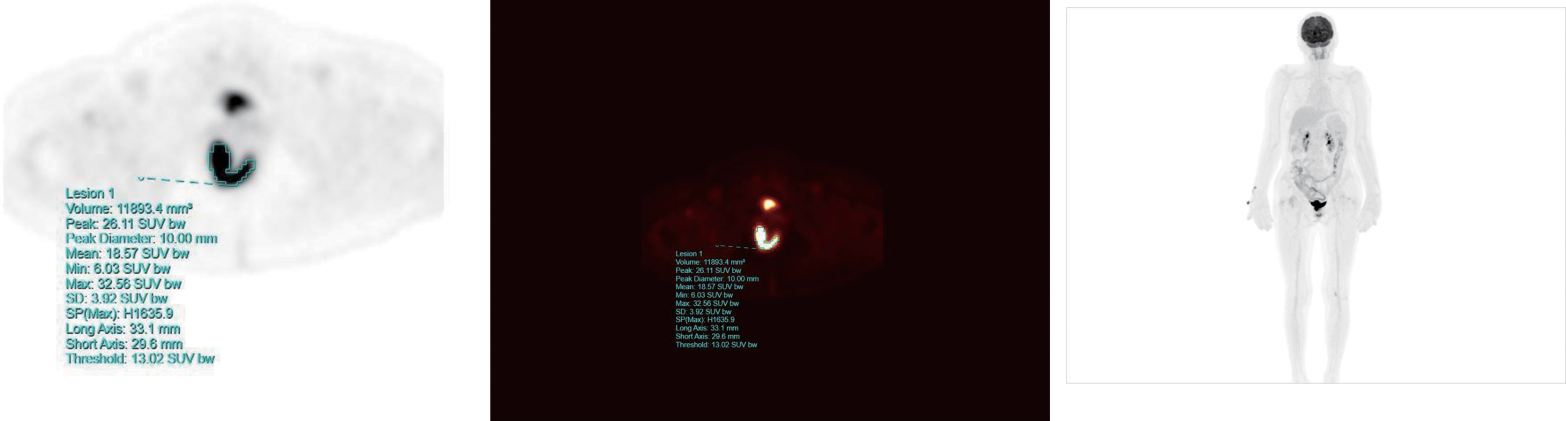
PET-CT image analysis and metabolic information calculation.

The acquired PET-CT original data were imported into the uWS-MR, UIH workstation. The images were processed and analysed by two senior physicians. In cases of disagreement, the result was obtained after discussion. The region of interest (ROI) of CRC lesions was delineated (single-layer ROI was placed, full-layer ROI was automatically traced and delineated, and manual correction was performed), as shown in Figure 1. SUVmax, SUVmean, SUVpeak, MTV, TLG and lymph node status (the current criteria for ^18^F-FDG PET/CT diagnosing regional lymph nodes metastasis in CRC were lymph node short diameter ≥1.0 cm or SUVmax ≥2.5) were measured and recorded.

### 2.5. PET-CT radiomics ROI delineation and feature extraction

**Figure 2:**
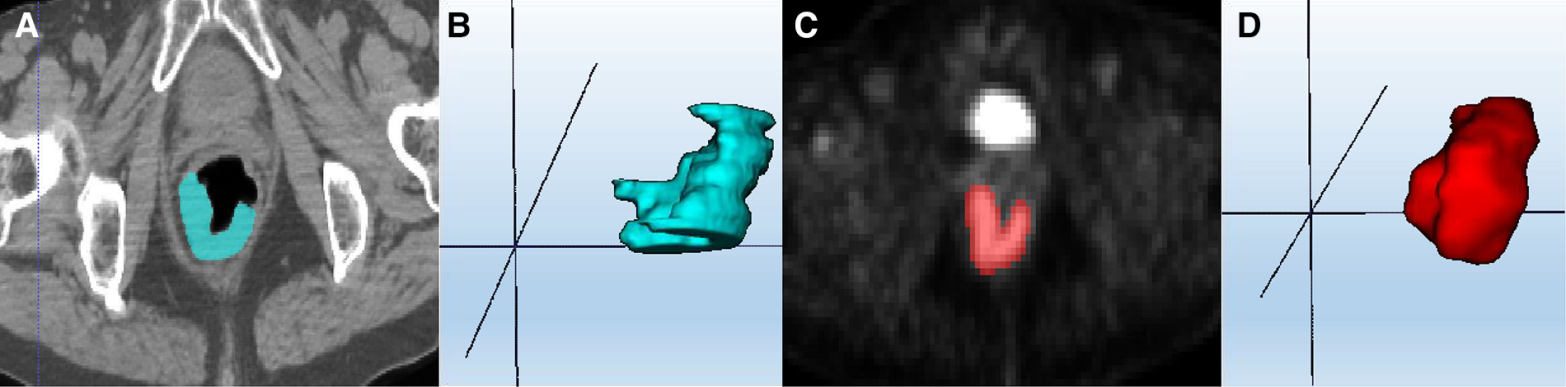
ROI segmentation of colorectal cancer lesions. (**A**) CT ROI segmentation; (**C**) PET ROI segmentation; (**B,D**) VOI based on multiple single-layer ROI fusion.

The original PET-CT images of all included patients were obtained from the MedEX System of Nuclear Medicine Workstation of Henan Provincial People's Hospital, and postprocessing was performed based on axial PET and CT sequences. Two radiologists with 5 years of experience applied the ITK-SNAP software (Version 3.8.0 http://www.itksnap.org/) to delineate the ROI layer by layer on rectal cancer lesions and obtain the tumour volume of interest (VOI), as shown in Figure 2. Finally, the VOI of each sequence was saved as a mask file.

Inter- and intra-class correlation coefficients (ICCs) were calculated for evaluation of the inter-reader reliability and intra-reader reproducibility of feature extraction. Radiologist 1 and radiologist 2 randomly selected 30 CT and PET images for radiomics ROI delineation and image feature extraction. Radiologist 1 repeated the segmentations 1 month later. An Intraclass correlation coefficient (ICC) greater than 0.75 indicated good agreement of feature extraction. The ROI segmentation for the remaining cases was performed by radiologist 1.

Radiomics features were calculated and combined for different imaging sequences (PET, CT). The open-source software PyRadiomics (Version 3.0.1, http://github.com/Radiomics/pyradiomics) was used feature extraction and analysis. Feature extraction was performed using filters such as Original, BoxMean and AdditiveGaussianNoise during preprocessing. First Order Statistics (Firstorder), Grey Level Co-occurrence Matrix (GLCM), Shape-based (3D) (Shape), Grey Level Dependence Matrix (GLDM), Neighbouring Grey Tone Difference Matrix (NGTDM), Grey Level Run-Length Matrix (GLRLM), and Grey Level Size Zone Matrix (GLSZM) features were generated from the ROI of image. Finally, 2,264 radiomics features were extracted from each sequence, and the PET-CT features set was generated by means of features stitching.

### 2.6. Feature selection and model establishment

The dataset was randomly divided into training and validation cohorts at a ratio of 6:4, the training cohort was used for features selection and models construction, and the test cohort was used for models’ evaluation. First, *Z* score normalization was performed on the extracted features, and then feature selection was performed on the preprocessed data, and the variance threshold method was used to remove the low variance features (*P *< 0.1). The features with low correlation with the classification label were removed based on the K-best method (*F* value method, *P *< 0.05). Finally, the irrelevant or redundant features were removed by the least absolute shrinkage and selection operator (LASSO), and the radiomics signature was constructed according to the weighted linear combination of the selected features and their corresponding regression coefficients. The radiomics score (Rad-score) of each patient was calculated accordingly. Finally, the training model is obtained based on logistic regression classifier.

Univariate logistic regression analysis was used to compare the differences in clinical information between the LVI-positive group and the LVI-negative group, and multivariate logistic regression analysis was used to identify the independent predictors significantly associated with LVI. Four datasets were constructed, and four prediction models were trained based on PET, CT radiomics features and clinical predictors. They were named PET_RS (PET radiomics features), CT_RS (CT radiomics features), PET-CT_RS (PET and CT radiomics features) and clinical radiomics model (with PET-CT radiomics features and clinical predictors).

### 2.7. Construction of radiomics nomogram, model prediction, and comparison

The predictive ability of the PET_RS, CT_RS, PET-CT_RS and the clinical radiomics models was evaluated based on the Area Under Curve (AUC), sensitivity, and specificity in the training and test cohorts. A radiomics nomogram was constructed by combining the clinical predictors of LVI screened by multivariate logistic regression and the Rad-score of the best model. The calibration of the nomogram was evaluated by drawing the calibration curve between the actual probability of LVI and the predicted probability of LVI. Decision curve analysis (DCA) was used to evaluate the clinical utility of multimodal radiomics by calculating the net benefit at different threshold probabilities.

### 2.8. Statistical analysis

All statistical analyses were performed using SPSS (version 26.0), R statistical software (version 4.0.2), and MedCalc (version 15.2.2). Measurement data following a normal distribution are represented as *x* ± *s*, and comparisons between the two groups were analysed using the two independent sample *t* test. Measurement data not following a normal distribution are represented as *M* (*P*25, *P*75), and comparisons between the two groups were analysed using the Mann‒Whitney *U* test. Count data are expressed as cases (%), and the chi-square test was used for comparisons between groups. Univariate and multivariate logistic regression methods (Forward selection) were used to analyse the clinical indicators of patients with CRC. The MedCalc software was used for the ICC test, receiver operating characteristic (ROC) curve drawing and analysis. The nomogram, calibration plots, and DCA were performed using R statistical software (Version 3.3.3, https://www.r-project.org). The “rms” package was used for nomogram and calibration curve drawing, and the “rmda” package was used for decision curve drawing. All statistical tests were two-sided, and *P *< 0.05 was considered statistically significant.

## 3. Results

### 3.1. Clinical characteristics

A total of 95 CRC patients were included in this study after screening, including 54 males and 41 females, with an average age of 62 years. None of the clinical factors significantly differed between the training cohort and the validation cohort (Table 1, *P* > 0.05). The above information indicates that the distribution of baseline clinical characteristics of patients in the training and validation cohorts was balanced. Among these patients, 44 (46.3%) were pathologically diagnosed to have LVI, and 51 (53.7%) were pathologically diagnosed to be free of LVI. A univariate logistic regression analysis of preoperative colorectal cancer LVI showed that maximum tumour diameter, lymph node metastasis, SUVmax and SUVmean metabolic values were independent risk factors for LVI (Table 2, *P *< 0.05). Multivariate logistic regression analysis finally included maximum tumour diameter, lymph node metastasis and SUVmean metabolism as independent clinical predictors of LVI (Table 3, *P *< 0.05).

**Table 1:** Clinical characteristics of CRC patients in the training and validation cohort.

Variables	Training cohort (*n* = 57)		Validation cohort (*n* = 38)		*X*^2^/*Z*/*t*	*P*
	LVI−	LVI+	LVI−	LVI+		
Age (years)					0.688	0.493
≤50	7 (53.8)	6 (46.2)	5 (55.6)	4 (44.4)		
>50	22 (50.0)	22 (50.0)	17 (58.6)	12 (41.4)		
Sex					0.064	0.800
Male	18 (54.5)	15 (45.5)	13 (61.9)	8 (38.1)		
Female	11 (45.8)	13 (54.2)	9 (52.9)	8 (47.0)		
Diameter (cm)					−0.135	0.893
≤3	12 (75.0)	4 (25.0)	9 (90.0)	1 (10.0)		
3–5	7 (36.8)	12 (63.2)	5 (50.0)	5 (50.0)		
>5	10 (45.5)	12 (54.5)	8 (44.4)	10 (55.6)		
Tumor grade					0.424	0.809
Well differentiated	2 (50.0)	2 (50.0)	3 (75.0)	1 (25.0)		
Middle differentiated	23 (53.5)	20 (46.5)	17 (56.7)	13 (43.3)		
Poorly differentiated	4 (40.0)	6 (60.0)	2 (50.0)	2 (50.0)		
CEA (ng/ml)					−1.091	0.275
≤5	19 (55.9)	15 (44.1)	18 (69.2)	8 (30.8)		
>5	10 (43.5)	13 (56.5)	4 (33.3)	8 (66.7)		
CA199 (U/ml)					−1.417	0.157
≤35	27 (57.4)	20 (42.6)	21 (61.8)	13 (38.2)		
>35	2 (20.0)	8 (80.0)	1 (25.0)	3 (75.0)		
CA125 (U/ml)					−0.259	0.796
≤35	28 (52.8)	25 (47.2)	22 (61.1)	14 (38.9)		
>35	1 (25.0)	3 (75.0)	0 (0.00)	2 (100.0)		
SUVmax (*x* ± *s*)	13.44 ± 6.18	14.05 ± 6.23	13.14 ± 5.31	14.91 ± 7.20	−0.112	0.911
SUVmean (*x* ± *s*)	7.50 ± 3.21	7.75 ± 3.21	7.36 ± 3.16	7.69 ± 3.84	0.188	0.851
SUVpeak (*x* ± *s*)	10.30 ± 4.91	10.99 ± 4.77	9.92 ± 4.03	11.26 ± 5.76	0.250	0.803
MTV (*M P*_25_, *P*_75_)	12.65 (8.13, 26.66)	14.00 (9.51, 19.12)	15.20 (8.36, 33.71)	16.68 (9.38, 20.93)	−0.642	0.521
TLG (*M P*_25_, *P*_75_)	95.27 (46.95, 189.95)	100.89 (61.65, 184.34)	113.48 (58.07, 194.30)	110.62 (46.92, 228.99)	−0.266	0.790
Lymph-node metastasis					0.001	1.000
Positive	14 (38.9)	22 (61.1)	10 (41.7)	14 (58.3)		
Negative	15 (71.4)	6 (28.6)	12 (85.7)	2 (14.3)		

SUVmax, maximum standardized uptake value; SUVmean, mean standardized uptake value; TLG, total lesion glycolysis; MTV, metabolic tumor volume; SUVpeak, standardized peak uptake; CEA, carcinoembryonic antigen; CA125, carbohydrate antigen 125; CA199, carbohydrate antigen199; diameter, maximum tumour diameter.

**Table 2:** Univariate logistic regression analysis of clinical predictors of LVI.

Variables	B	SE	Wald	*P* value	Exp (B)	95.0% *CI* for Exp (B)	
						Lower	Upper
Age	−0.001	0.016	0.007	0.931	0.999	0.968	1.031
Sex	0.347	0.416	0.696	0.404	1.415	0.626	3.201
Diameter	0.410	0.137	8.929	0.003^a^	1.507	1.152	1.972
CEA	0.001	0.002	0.544	0.461	1.001	0.998	1.005
CA199	0.004	0.004	1.380	0.240	1.004	0.997	1.012
CA125	0.023	0.017	1.818	0.178	1.023	0.990	1.058
SUVmax	1.113	0.040	8.056	0.005^a^	1.120	1.036	1.211
SUVmean	0.252	0.075	11.340	0.001^a^	1.287	1.111	1.490
MTV	0.000	0.009	0.002	0.967	1.000	0.983	1.018
TLG	0.001	0.001	0.491	0.483	1.001	0.999	1.003
SUVpeak	0.038	0.044	0.744	0.388	1.039	0.952	1.134
Lymph-node metastasis	1.622	0.481	11.363	0.001^a^	5.062	1.972	12.999

SUVmax, maximum standardized uptake value; SUVmean, mean standardized uptake value; TLG, total lesion glycolysis; MTV, metabolic tumor volume; SUVpeak, standardized peak uptake; CEA, carcinoembryonic antigen; CA125, carbohydrate antigen 125; CA199, carbohydrate antigen199; diameter, maximum tumour diameter.

^a^ These variables were statistically significant for predicting LVI status in the univariate analyses.

**Table 3:** Multivariate logistic regression analysis of clinical predictors of LVI.

Variables	B	SE	Wald	*P* value	Exp (B)	95.0% *CI* for Exp (B)	
						Lower	Upper
SUVmax	−0.183	0.112	2.655	0.103	0.833	0.669	1.038
SUVmean	0.598	0.222	7.251	0.007^a^	1.819	1.177	2.811
Diameter	0.436	0.151	8.335	0.004^a^	1.546	1.150	2.078
Lymph-node metastasis	1.468	0.566	6.720	0.010^a^	4.339	1.430	13.162

SUVmax, maximum standardized uptake value; SUVmean, mean standardized uptake value; diameter, maximum tumour diameter.

^a^ These variables that were statistically significant for predicting LVI status in the multivariate analysis.

### 3.2. Radiomics analysis

**Figure 3:**
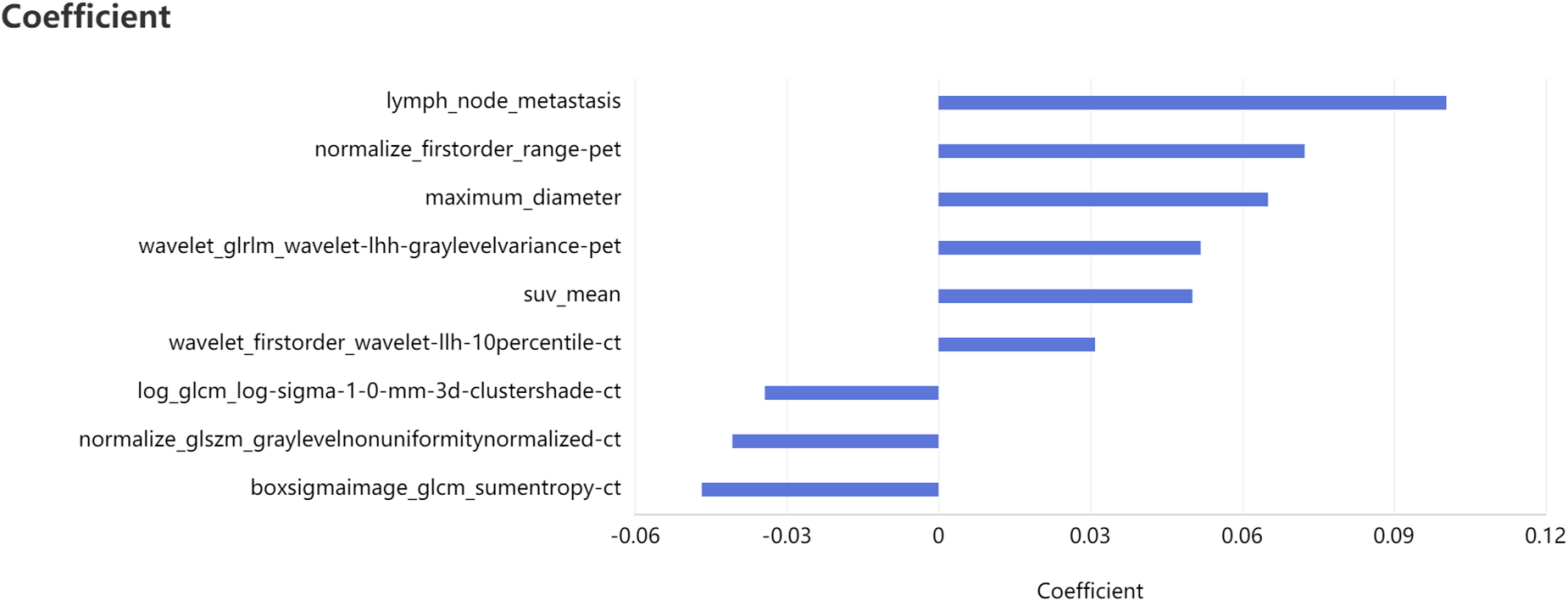
Feature selection of clinical radiomics model four CT features, two PET features, three clinical features and their correlation coefficients were selected after feature dimension reduction.

**Figure 4:**
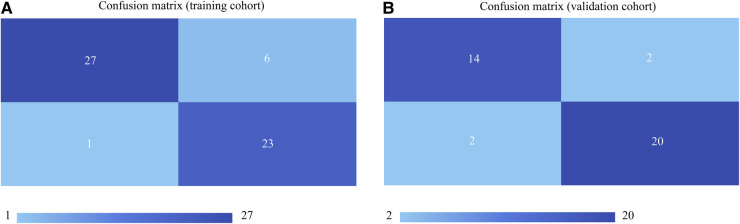
Confusion matrices of the models in the training cohort (**A**) and validation cohort (**B**) the upper left and lower right corners represent true positive and true negative, respectively, and the upper right and lower left corners represent false positive and false negative, respectively. The larger the true positive and true negative values, the better the model performance.

Taking the clinical radiomics model as an example, firstly, to avoid subjective differences in segmentation of ROI, the radiomics features with both inter- and intra-reader ICCs > 0.75 were retained. Secondly, two PET features and four CT features were selected by variance threshold, K-best and LASSO regression models, and then combined with three clinical predictors, a total of nine features were obtained. The selected features were summed by weighting coefficients to calculate the Rad-score (Figure 3). Finally, the best prediction model was established based on the logistic regression classifier. The Rad-score significantly differed between LVI-positive and LVI-negative patients (*P* < 0.001). The confusion matrix shown in Figure 4 shows the prediction performance of the training and test set models.

### 3.3. Assessment and validation of prediction models for LVI status

**Figure 5:**
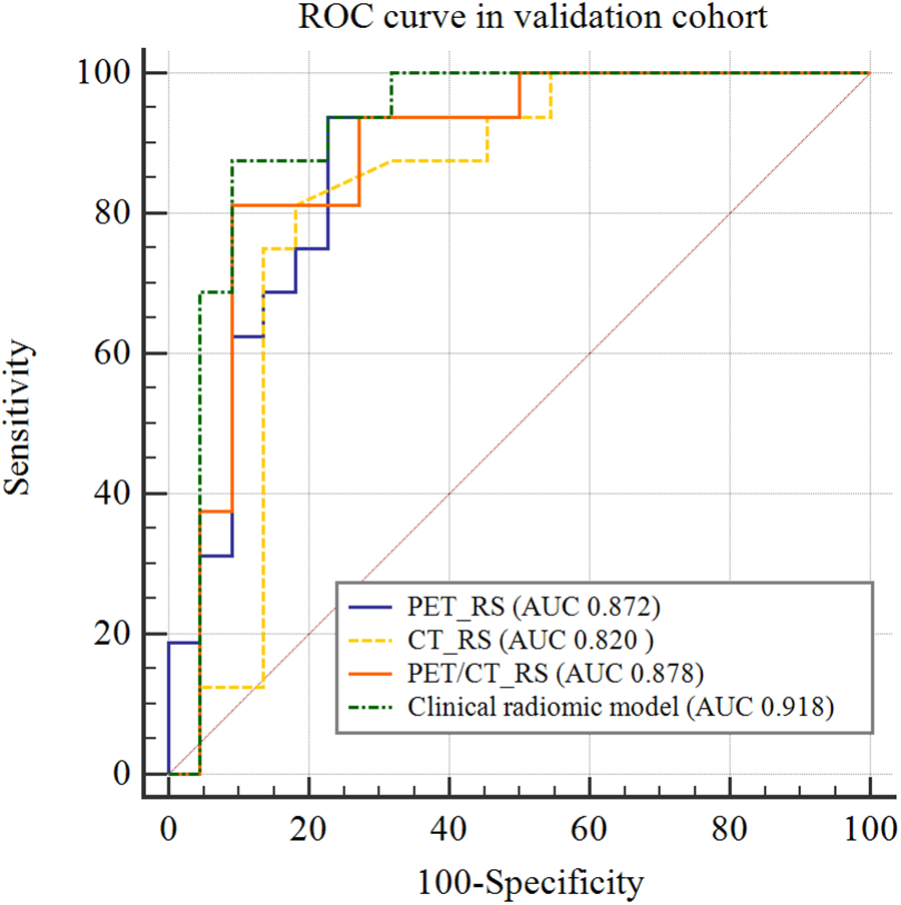
Comparison of receiver operating characteristics (ROC) curves for predicting lymphovascular invasion in the validation cohort.

**Figure 6:**
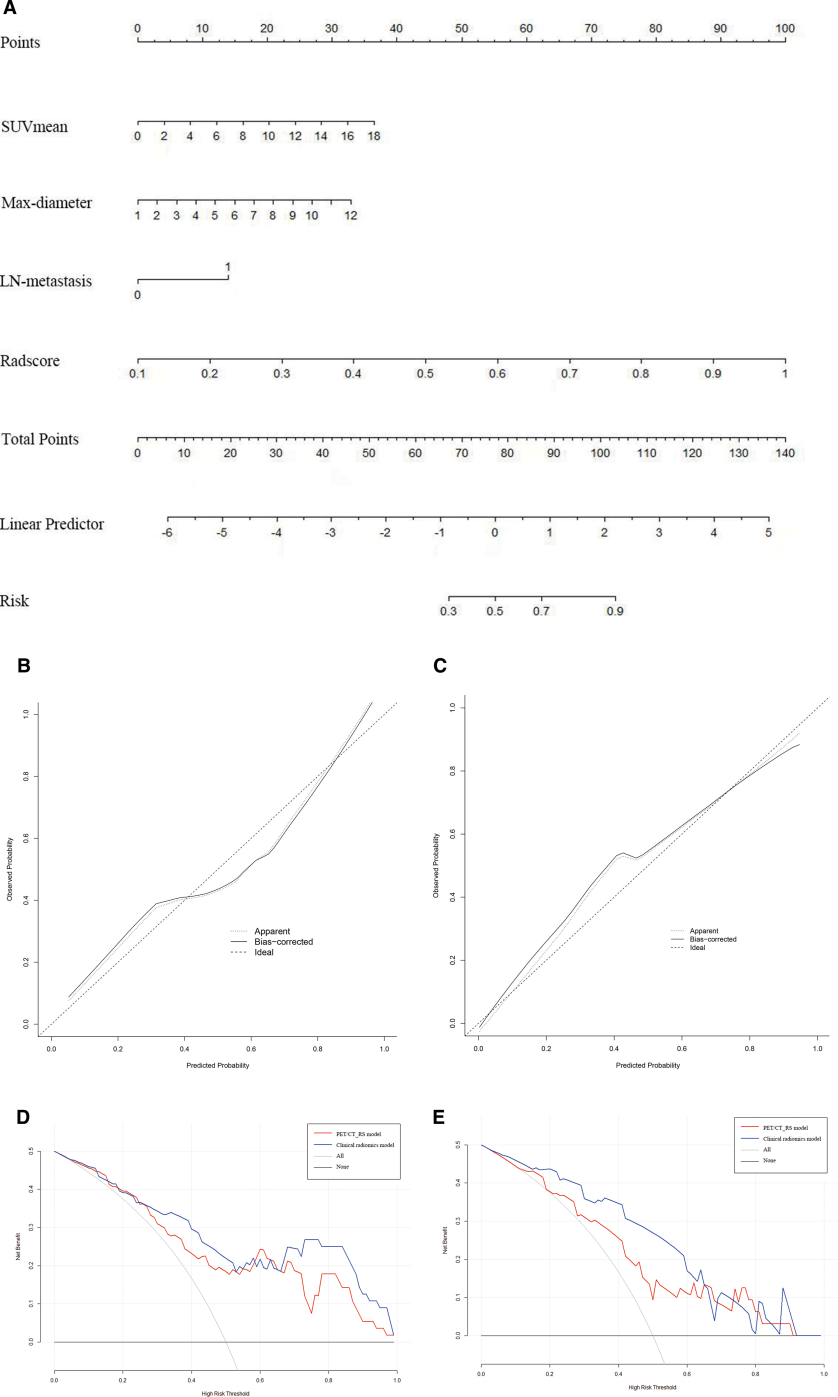
(**A**) Nomogram of clinical radiomics model developed based on logistic regression model for training cohort. Max-diameter, Maximum tumour diameter; LN-metastasis: Lymph node metastasis. (**B,C**) Calibration curves of the nomogram in the training (**B**) and validation cohorts (**C**). The apparent line indicates the performance of the nomogram, the closer it is to the solid diagonal line, the better the prediction. (**D,E**) Decision curve analysis (DCA) of prediction model in the training (**D**) and validation cohorts (**E**).

By comparing the area under the ROC curve (AUC), sensitivity, and specificity of each model (Table 4 and Figure 5), the clinical radiomics model (AUC: 0.918, 95% CI 0.782, 0.982) had the best performance, with high sensitivity (87.5%) and specificity (90.9%), which was confirmed to be the best prediction model. We generated a visual nomogram based on the training cohort of the clinical radiomics model (Figure 6A). The calibration curves of both the training cohort and the validation cohort of the nomogram demonstrated good agreement between the predicted LVI and the actual observation (Figures 6B,C). The decision curves of the PET-CT_RS and clinical radiomics models are shown in Figures 6D,E. The results showed that the clinical radiomics model yielded a higher overall net benefit than the PET-CT_RS model.

**Table 4:** Diagnostic efficacy of PET/CT omics model and combined model for vascular invasion.

Model	Training cohort (*n* = 57)				Validation cohort (*n* = 38)			
	AUC (95% CI)	Sensitivity (%)	Specifcity (%)	Youden index	AUC (95% CI)	Sensitivity (%)	Specifcity (%)	Youden index
PET_RS	0.764 [0.632, 0.866]	82.14	58.62	0.41	0.872 [0.724, 0.958]	93.75	77.27	0.71
CT_RS	0.964 [0.878, 0.996]	90.00	93.10	0.93	0.820 [0.661, 0.925]	81.25	81.82	0.63
PET-CT_RS	0.932 [0.833, 0.982]	92.86	79.31	0.72	0.878 [0.731, 0.961]	81.25	90.91	0.72
Clinical radiomics model	0.922 [0.820, 0.977]	96.43	79.31	0.72	0.918 [0.782, 0.982]	87.50	90.91	0.78

PET-RS: only PET radiomics features; CT-RS: only CT radiomics features; PET-CT_RS: PET and CT radiomics features; clinical radiomics model: with PET-CT radiomics features and clinical predictors.

## 4. Discussion

In this study, PET-CT radiomics features and clinical predictors were used to construct a clinical radiomics model for preoperative prediction of pathological LVI in CRC. We found that the clinical radiomics nomogram integrating PET-CT radiomics score, SUVmean, maximum tumour diameter, and lymph node status had good predictive value for LVI in CRC and could assist clinical decision-making. Clinical factors add additional value to radiomics in predicting LVI, and it was expected to become a biomarker to assist clinical decision making in CRC patients.

LVI refers to the invasion of cancer cells into the surrounding tissues or even the entire body through veins or small vessels (15). LVI is not only a subclinical marker of lymph node metastasis but also a high-risk factor for colorectal cancer recurrence and metastasis. The prognosis and overall survival rate of colorectal cancer patients with transvascular invasion are significantly worse than those of colorectal cancer patients without invasion (16–18). Therefore, studying the presence of vascular invasion is of great clinical value. Unlike recognition with the naked eye used in traditional imaging methods, radiomics analysis automatically screens image features and studies tumour heterogeneity in depth. In addition, this study used manual layer-by-layer delineation of 3D ROI, which may contain more important spatial features than conventional single-layer image delineation (2D ROI) (19). The central hypothesis (20) of the development of radiomics research is to describe the tumour microenvironment based on radiomics methods to assist in the evaluation of the biological characteristics of tumours, and LVI is one of the important components of the tumour microenvironment. Previous studies have explored the important clinical value of radiomics analysis in predicting pathological LVI. Zhang et al. (21) found that a multimodal radiomics model constructed by multisequence MR and contrast-enhanced CT can be used as an effective visual prognostic tool for predicting LVI in rectal cancer, and its preoperative prediction shows great potential in improving treatment decisions. Kim et al. (22) used pelvic magnetic resonance imaging to assess preoperative LVI in patients with rectal cancer, which had good specificity (93.2%) but low sensitivity (68.2%). PET/MRI can simultaneously provide metabolic, diffusion and perfusion information with high specificity (86.2%) and sensitivity (80.0%), which is helpful to predict LVI more accurately (23).

Consistent with the results of many previous studies, combining clinical risk factors can improve the performance of prediction models (24–26). The clinical factors (lymph node status and maximum tumour diameter) and metabolic parameters (SUVmean) screened in this study played a complementary role in predicting LVI. Previous studies have demonstrated that baseline metabolic parameters play an important role in predicting tumour LVI. Hyun et al. (27) reported that the tumour-liver standardized uptake value ratio was closely related to the occurrence of microvascular invasion. In the study by Yang et al. (28), the maximum standardized uptake value (SUVmax) was considered to be an independent predictor of LVI in gastric cancer. In our study, univariate logistic regression analysis showed that both SUVmax and SUVmean were independent risk factors for LVI, However, SUVmax had a low weight in the clinical radiomics model and was not included in the final model after multivariate logistic regression analysis.

SUVmax is calculated based on the maximum voxel grey value and is the most commonly used metabolic parameter in PET-CT. It is not affected by the size of the ROI and can be used to initially differentiate benign and malignant tumours. However, it only represents the maximum metabolic value of the tumour and reflects the highest activity of the lesion but cannot reflect the overall characteristics of the tumour. Multiple studies (29–31) have found that SUVmean refers to the average SUV of ROI, which is less susceptible to fluctuations in counting statistics and ROI placement and is more representative of tumour lesion metabolism, similar to the results of this study. MTV was defined as the sum of all voxels with abnormal drug uptake in the ROI. The MTV is generally calculated using the percentage threshold method, but a single percentage threshold will cause the measured MTV value to be too large or too small. TLG is the product of the MTV of the lesion and SUVmean in the same volume, which can reflect both the metabolic activity and metabolic volume of the tumour. In this study, MTV and TLG were not found to have good predictive efficacy, which was speculated to be due to the difference in threshold setting. Abelson et al. (32) studied whether ^18^F-FDG PET-CT imaging metrics were associated with prognosis in patients with non-small cell lung cancer undergoing stereotactic ablative radiation therapy, and they reported that metabolic tumour volumes with pretreatment MTV values higher than SUV 7 or 10 could be used to predict overall survival, whereas metabolic tumour volumes with low thresholds SUV 2 and SUV 4) were not associated with overall survival. However, our study used a lower SUV threshold of 4.0 (median SUV). Although TLG can reflect both tumour metabolism and tumour volume, TLG and LVI did not significantly correlate in this study, which may be due to different thresholds for calculating MTV.

In addition, we concluded the presence or absence of lymph nodes with abnormal metabolism is also one of the predictors of LVI. Although the lymph node status determined by PET-CT is not as accurate as that determined by surgical pathology, it reflects the overall situation of lymph node metastasis to a certain extent. Colorectal cancer lesions with positive lymph node metastasis are likely to have microscopically invaded the lymphovascular tissue around the intestine, which is consistent with the findings of Aktekin et al. (33). In addition, our study found that the maximum diameter of the tumour was also one of the predictors of LVI in rectal cancer, which also confirmed the research results of Chen et al. (34, 35): larger tumours and deeper invasion depths increased the probability that the tumour will invade the surrounding lymph and vascular tissues. Therefore, for patients with a larger tumour volume and deeper invasion degree found during surgery, the surgical plan should be carefully considered, and the resection should be extended if necessary. Other conventional clinical indicators, such as age, sex, and tumour chemical markers (CEA, CA199, and CA125), showed different results in different studies. Li et al. (36) showed that the above clinical indicators were not included in the multivariate regression model of vascular invasion of rectal cancer, which was consistent with the results of this study. However, some studies have shown that CEA is of great significance in the prognostic evaluation of CRC patients with nerve and vascular invasion (37, 38). This finding is not consistent with the results of this study, which may be related to the different research methods or sample sizes and inclusion and exclusion criteria.

## 5. Limitations

Our study has some limitations. First, due to the strict inclusion and exclusion criteria, the sample size was small, and we will continue to expand the sample size for further research in the future. Second, this study was a single-centre study and lacked independent external validation. We plan to include multicentre samples in future studies to address these issues.

## 6. Conclusions

In summary, based on PET-CT imaging omics combined with clinical risk prediction factors, a clinical radiomics prediction model for the prediction of LVI was developed. The model has good diagnostic performance and can be used as an individualized decision support tool to evaluate the preoperative LVI status of colorectal cancer patients, which can better assist clinicians to make preoperative decision-making, postoperative evaluation, and formulate individualized clinical treatment plans for patients.

## Data Availability

The original contributions presented in the study are included in the article/Supplementary Material, further inquiries can be directed to the corresponding author.
